# Feasibility and Validity of a Standardized Protocol for Measuring Thoracic Aortic Aneurysm Morphology

**DOI:** 10.1177/15266028251380535

**Published:** 2025-10-05

**Authors:** Olivier L.R.M. van Tongeren, Amber Sharman, Vinamr Rastogi, Rens B. Varkevisser, Sanne E. Hoeks, Arnoud V. Kamman, Hence J.M. Verhagen, Jorg L. de Bruin

**Affiliations:** 1 Department of Vascular Surgery, Erasmus University Medical Centre, Rotterdam, The Netherlands; 2 Division of Vascular and Endovascular Surgery, Department of Surgery, Beth Israel Deaconess Medical Center, Boston, MA, USA; 3 Department of Anaesthesiology, Erasmus University Medical Centre, Rotterdam, The Netherlands

**Keywords:** thoracic aortic aneurysm, thoracic endovascular aortic repair, standardized measurement protocol, computed tomography angiography

## Abstract

**Introduction:**

Thoracic endovascular aortic repair (TEVAR) is the predominant treatment for thoracic aortic aneurysms (TAA) due to its superior perioperative outcomes. International guidelines recommend assessing TAA characteristics via computed tomography angiography (CTA). However, the lack of a standardized measurements protocol introduces variability in preoperative planning, and imaging surveillance, and therefore hinders artificial intelligence (AI) integration for fully automated measurements. This study aims to develop and validate a standardized measurement protocol for TAA to improve consistency in measurement and enhance imaging surveillance.

**Methods:**

A retrospective cohort study was performed at a Dutch tertiary center on patients who underwent TEVAR for a descending TAA from 2010 to 2019. We included degenerative and mycotic TAAs, and exclusions were the lack of a preoperative CTA and/or incomplete postoperative imaging. A standardized measurement protocol was developed based on expert opinion and validated endovascular aortic repair (EVAR) protocols. Imaging analysis utilized dedicated 3D imaging software. The protocol included semi-automated 3D segmentation, center lumen line (CLL) reconstruction, and several measurements, including aortic diameter/volume and sealing lengths. Intraobserver and interobserver agreements were assessed using Bland-Altman analysis and intraclass correlation coefficients (ICCs).

**Results:**

We analyzed 133 CTA scans from 31 patients, showing high levels of agreement across measurements, particularly for those repeated by the same observer. Maximum diameter measurements demonstrated excellent consistency, with minimal mean differences for intraobserver and interobserver agreements and excellent correlation (ICC>0.900). Volume measurements were similar consistent, with mean differences of 3.45 (intraobserver) and 2.75 (interobserver) cc. Proximal and distal seal measurements showed good agreement, although interobserver correlation was slightly less consistent. Length of coverage by the endograft exhibited strong consistency.

**Conclusion:**

Our standardized measurement protocol for descending TAA offers a consistent approach for preoperative planning, imaging surveillance, and research applications, with high agreement in most measurements. This consistency could reduce variability, enhance imaging surveillance. Future research should focus on external validation, integrating AI to further improve measurement consistency, and simplify TEVAR surveillance.

**Clinical Impact:**

This retrospective study of 133 CTA scans from 31 thoracic endovascular aortic repair (TEVAR) for TAA patients demonstrated excellent measurements consistency, including maximum diameter showing strong agreement and minimal differences in volume measurements. In the absence of an existing measurements protocol, our proposed standardized protocol supports improved preoperative planning, imaging surveillance to further enhance TEVAR surveillance.

## Introduction

Thoracic endovascular aortic repair (TEVAR) is the preferred treatment modality for descending thoracic aortic aneurysm (TAA) due to superior perioperative outcomes over open repair.^1–4^ For TEVAR planning, sizing, and follow-up, computed tomography angiography (CTA) imaging is recommended by current European Society for Vascular Surgery (ESVS) and Society for Vascular Surgery’s (SVS) guidelines.^5,6^ Primary clinical success includes the absence of aneurysm expansion (diameter>5 mm or volume>10%), while failure includes graft migration, endoleaks, and aneurysm expansion.^7^

Despite this importance, no universally standardized measurement protocol exists for descending TAA.^8^ Current international guidelines provide recommendations on reporting and imaging follow-up, but largely leave the measurement process itself to clinical practice, resulting in interobserver and interinstitutional variabilities.^5–7,9^ This lack of consensus has resulted in notable heterogeneity in measurement protocols among tertiary centers specializing in aortic disease.^10^ In contrast, validated protocols for endovascular aortic repair (EVAR)^11,12^ ensure consistency in measurements and outcomes. Adopting a standardized protocol for TAA could improve preoperative sizing, enhance postoperative imaging surveillance, and facilitate benchmarking across centers.

Moreover, with emerging artificial intelligence (AI) applications to improve and greatly facilitating TEVAR surveillance,^13^ standardization is imperative for reliable integration into clinical practice. Therefore, this study aimed to compose and internally evaluate the consistency of a newly developed standardized measurement protocol following TEVAR for descending TAA.

## Materials and Methods

### Study Design and Patient Population

**Figure 1.**
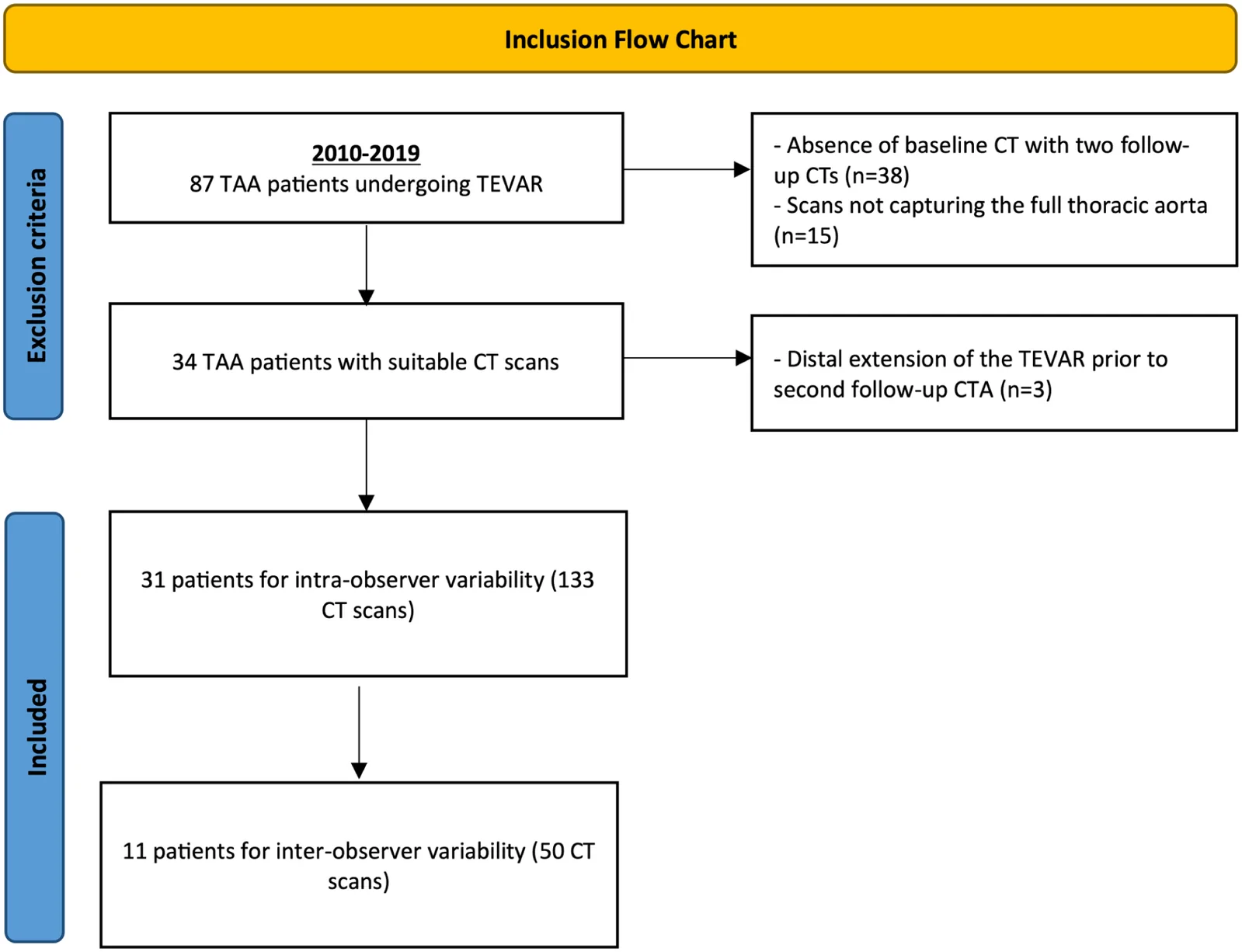
Inclusion diagram. TEVAR, thoracic endovascular aneurysm repair.

We conducted a retrospective cohort study of patients who underwent TEVAR for isolated descending TAA, at a single tertiary center in the Netherlands between 2010 and 2019 (n=87; Figure 1). Only degenerative and mycotic TAAs were included. To ensure availability of at least 2 postoperative CTA scans, the study period was limited to patients treated up to 2019. Nevertheless, patients were excluded if they lacked a preoperative baseline CTA scan accompanied by 2 follow-up CTA scans (n=38). For this study, we required a complete imaging of the thoracic aorta, thus patients with a scan that did not encompass the entire thoracic aorta were excluded (n=15). In addition, patients who received a distal extension of the TEVAR prior to the second follow-up CTA (n=3) were excluded. All CTAs for the included patients had a maximum slice thickness of 3 mm. The study protocol received approval from the hospital’s institutional and ethical review board and given its retrospective design, patient informed consent was not required (MEC-2022-0591). This study was conducted in accordance with the STROBE (strengthening the reporting of observational studies in epidemiology) criteria.^14^

### Data Collection and Image Analysis

We extracted patient baseline demographics and comorbid conditions from the hospital’s electronic patient records software (ChipSoft HIX, Amsterdam, The Netherlands). Variables were defined in accordance with the reporting standards for TAA repair.^7^ All CTA scans were performed on a dual-source CT scanner (SOMATOM Drive or Force, Siemens Healthineers, Erlangen, Germany). Aneurysm imaging analyses were performed using 3mensio Vascular® 4.2 reconstruction software (Pie medical, Bilthoven, The Netherlands).

### Proposed Measurement Protocol

**Figure 2.**
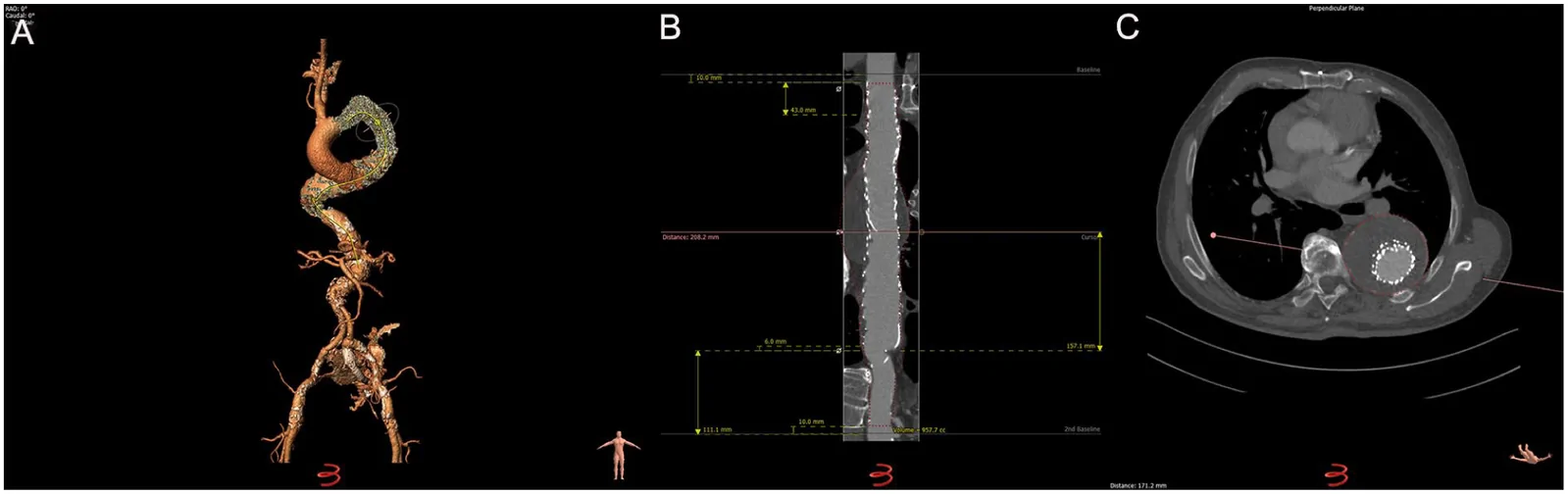
Features of the proposed measurement protocol. (A) Three-dimensional segmentation of the aorta with center lumen lines (CLLs) drawn. (B) Stretched vessel view used for volume measurements. (C) Perpendicular axial view used for diameter measurements.

The rationale behind the protocol was based on expert opinion, guidelines and previously validated protocols for surveillance after EVAR.^7,11,12^ The features of the protocol are provided in Figure 2. After semi-automated 3D segmentation of the aorta, a center lumen line (CLL) was established, spanning from the ascending aorta to the level of the renal arteries (Figure 2A). Multiplanar reformatted images (MPRs) were acquired, displaying the aorta in both parallel (vessel stretched; Figure 2B) and perpendicular (orthogonal) views. To ensure consistent measurement levels, 2 baselines were manually established on the stretched vessel view, marking 2 anatomical landmarks (Figure 2C). The first baseline was defined starting in the aorta from the center of the brachiocephalic trunk, while the second baseline was positioned just proximal to the highest renal artery.

Aortic length was measured along the CLL in the stretched vessel view as the distance between the baselines.

Maximum aortic diameter (outer-to-outer diameter) was calculated by measuring 2 diameters, perpendicular to the CLL, at the point of maximum dilation. When the difference between these perpendicular measurements exceeded 5%, the smaller diameter was recorded as the maximum diameter to avoid overestimation due to vessel tortuosity (Figure 2C).^15^ When the difference was less than 5%, indicating a near-circular shape, the maximum diameter was defined as the average of the perpendicular measurements. For saccular aneurysms, only the largest diameter in CLL view was recorded and consistently used for subsequent CTAs of the same patient.

Aortic volume was measured by manually creating the outer-vessel-line on the stretched vessel view in 4 dimensions from 10 mm below the first baseline to 10 mm above the second baseline. Subsequently, aortic volume was automatically calculated using a function provided by 3mensio.

Proximal and distal diameters at the level of the seal were measured perpendicular to the CLL, using outer-to-outer diameter, regardless of whether the endograft had already been placed.^15^ Each diameter was determined as the average of the 2 perpendicular measurements. The distance to the proximal and distal end of the fabric was measured perpendicularly to their respective baselines. Proximal and distal lengths of sealing were quantified.

In addition, the zones in which the proximal and distal ends of the endograft sealed, known as the proximal and distal zones of attachment, were identified and described according to the guidelines.^7^ Finally, the length of coverage by the endograft was measured using a custom length tool provided by the software.

### Definitions and Variables

The primary outcome included the consistency of the proposed protocol, assessed by both intraobserver and interobserver agreements. Both observers were extensively trained in vascular imaging, and the measurements were performed under the supervision of experienced vascular surgeons. The intraobserver agreement was assessed by a single observer (a senior medical student) who performed duplicate measurements on the same subjects and CTA scans, with a minimum interval of 4 weeks between measurements to prevent recall bias. Interobserver agreement was evaluated by a second observer (a vascular surgery research fellow) who independently analyzed a randomly selected subgroup of patients, without looking at any patient details prior to the selection. Both observers were experienced and have assessed multiple EVAR, TEVAR, and F/BEVAR cases before.

### Statistical Analysis

We reported categorical variables as counts and percentages, while continuous variables were described using the median accompanied by the interquartile range (IQR). Normal distribution was determined using visual inspection with histograms and Q-Q plots and the Shapiro-Wilk test. In our study, 95% confidence intervals (CIs) were utilized, and a p value of less than 0.05 was considered as statistically significant.

Intraobserver and interobserver agreements were evaluated using Bland-Altman analysis (B-A plot) and the intraclass correlation coefficient (ICC).^16–18^ This analysis provides both the mean difference (bias) and the percentage of mean difference relative to the average for each measurement variable. A minimal mean difference indicates a minimal variation between observers or across repeated measurements. The limits of agreement (LoA) were defined as the mean bias ± 1.96 times standard deviation (SD), which outlines the range where 95% of the differences between observers would be expected to fall. Heteroscedasticity was determined using visual inspection. In the case of heteroscedasticity, we applied a logarithmic transformation to the values alongside the B-A method for the 95% confidence limits calculation. These limits were then back transformed to facilitate interpretation of the agreement. As a subgroup analysis, we assessed whether intraobserver and interobserver differences varied between low (0.8 and 1.0 mm) and high (1.5, 2.0, and 3.0 mm) CTA slice thickness, we used either a 2-sample *t* test or a Mann-Whitney *U* test for each measurement, depending on data distribution.

Furthermore, for assessing intraobserver agreement, we employed a 2-way mixed effects, single-rater ICC model, whereas for interobserver agreement, we used a 2-way random effects, single-rater ICC model.^18^ The ICC values were interpreted using the cut-off threshold defined by Landis and Koch.^19^

All statistical processes were executed using R version 4.3.2 (http://www.r-project.org).

## Results

### Baseline Characteristics

A total of 31 patients who underwent TEVAR for TAA were included in the analysis (Figure 2), contributing to a data set of 133 CTA scans. The intraobserver agreement analysis utilized data from the entire cohort, while the interobserver agreement was based on 50 CTA scans from 11 randomly selected patients from the entire cohort. The median age of the cohort was 72 years (IQR: 68.0, 75.0; Table 1), with a predominance of male patients (67.7%). We included 5 levels of CTA slice thickness, with had a median of 1.0 (IQR: 0.8–3.0) mm. Additional demographics and clinical variables are detailed in Table 1.

**Table 1.** Baseline Characteristics of Total Cohort.

	Total cohort
	(N=31)
Age, median (IQR)	72.0 (68.0, 75.0)
Male sex	21 (67.7)
Myocardial infarction	5 (16.1)
Diabetes mellitus	4 (12.9)
Hypertension	24 (77.4)
Peripheral artery disease	14 (45.2)
History of cancer	7 (22.6)
Pulmonary disease	9 (29.0)
*eGFR*	
*<30*	3 (9.7)
*30–60*	20 (64.5)
*>60*	8 (25.8)
Smoking status	
*Non-smoker*	3 (9.7)
*Current smoker*	9 (29.0)
*Former smoker*	14 (45.2)
Anticoagulant	7 (22.6)
Antiplatelet	17 (54.8)
Statin	26 (83.9)
Beta blocker	19 (61.3)
Timing of surgery	
*Elective*	23 (74.2)
*Symptomatic*	4 (12.9)
*Ruptured*	4 (12.9)
Device modality	
*Gore*	7 (22.6)
*Medtronic valiant*	11 (35.5)
*Other*	10 (32.3)

Data were presented as counts with percentages (%) or medians with interquartile ranges (IQRs).

Abbreviations: eGFR, estimated glomerular filtration rate; IQR, interquartile range.

### Diameter and Volume Measurements

**Figure 3.**
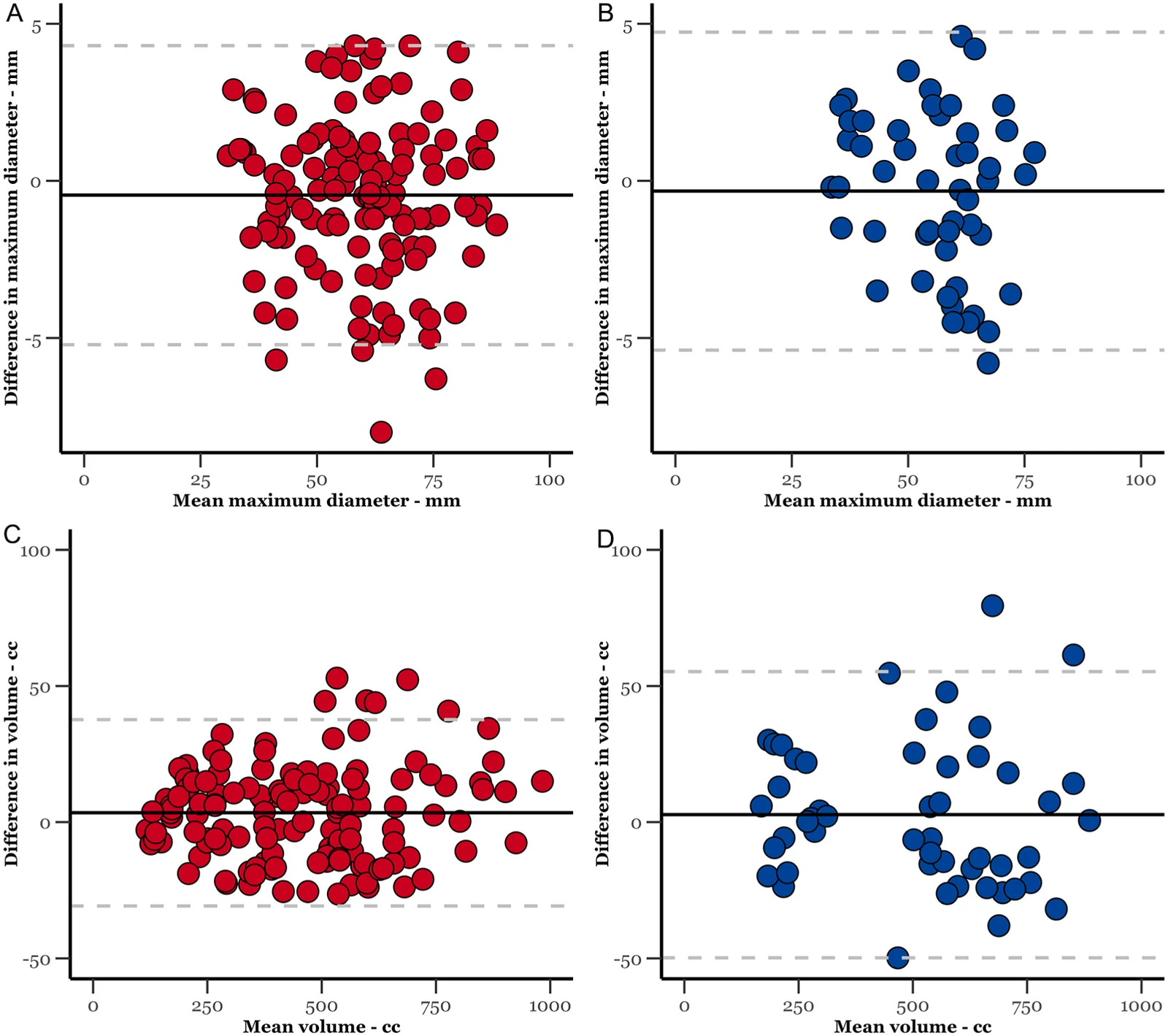
Bland-Altman plot of the intraobserver (in red) and interobserver (in blue) agreements for thoracic aneurysm diameter and volume.

The intraobserver and interobserver agreement results for all variables are presented in Table 2. For maximal aneurysm diameter, B-A analysis demonstrated minimal variation between observers and across repeated measurements. The mean intraobserver difference was −0.45 mm (–0.77%; LoA: –5.21, 4.30 mm), meaning the same observer’s repeated measurements differed by an average of 0.45 mm. The mean interobserver difference was −0.33 mm (–0.58%; LoA: –5.39, 4.73 mm)(Figure 3). These findings reflect strong consistency in diameter measurements.

**Table 2.** Overview of the Intraobserver and Interobserver Agreement Outcomes.

	Mean	Mean bias (percentage of the mean)	95% limits of agreement
*Intraobserver*			
Maximal diameter (mm)	59.18	−0.45 (−0.77%)	−5.21, 4.30
TAA volume (cc)	450.44	3.45 (0.77%)	−30.75, 37.66
Length of coverage (mm)	156.75	1.68 (1.07%)	−14.08, 17.43
TAA length (mm)	373.53	2.52 (0.67%)	−15.35, 20.38
Diameter proximal seal (mm)	33.43	0.23 (0.67%)	−3.10, 3.55
Distance to proximal seal (mm)	63.23	−0.16 (−0.26%)	−10.55, 10.22
Diameter distal seal (mm)	32.81	0.40 (1.23%)	−1.89, 2.69
Distance to distal seal (mm)	138.11	0.32 (0.23%)	−8.45, 9.09
Length of proximal seal (mm)	19.77	0.84 (4.25%)	−6.33, 8.01
Length of distal seal (mm)	27.53	−0.02 (−0.09%)	−6.52, 6.47
*Interobserver*			
Maximal diameter (mm)	55.86	−0.33 (−0.58%)	−5.39, 4.73
TAA volume (cc)	502.76	2.75 (0.55%)	−49.80, 55.30
Length of coverage (mm)	177.81	6.35 (3.57%)	−9.15, 21.86
TAA length (mm)	384.09	7.39 (1.93%)	−13.10, 27.89
Diameter proximal seal (mm)	34.19	−1.94 (−5.66%)	−6.33, 2.46
Distance to proximal seal (mm)	67.16	2.32 (3.46%)	−10.89, 15.54
Diameter distal seal (mm)	36.29	1.05^a^ (2.91%)	0.92, 1.21^a^
Distance to distal seal (mm)	122.97	−0.18 (−0.15%)	−14.41, 14.05
Length of proximal seal (mm)	19.40	2.61 (13.44%)	−23.85, 29.06
Length of distal seal (mm)	27.35	8.15 (29.80)	−18.56, 34.86

Abbreviations: TAA, thoracic aortic aneurysm.

a Following log-back transformation.

Aneurysm volume measurements demonstrated similarly consistent results. Intraobserver agreement showed a mean difference of 3.45 cc (0.77%; LoA: –30.75, 37.66), while interobserver agreement showed a mean difference of 2.75 cc (0.55%; LoA: –49.80, 55.30).

### Proximal Seal Diameter and Distance to End of Fabric Measurements

**Figure 4.**
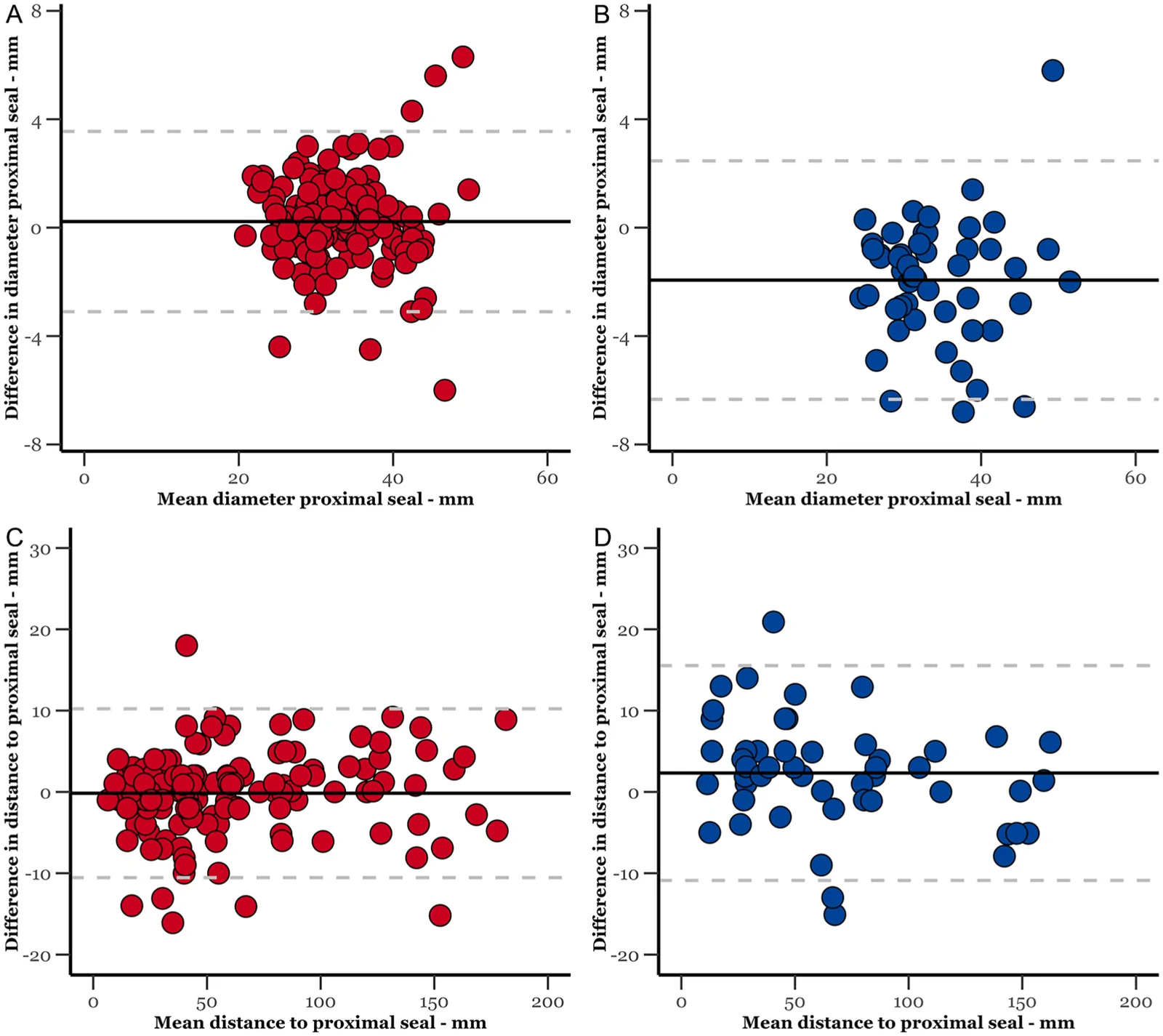
Bland-Altman plot of the intraobserver (in red) and interobserver (in blue) agreements for diameter and distance proximal seal.

Intraobserver and interobserver agreements were strong for both the diameter of the proximal seal and the distance to the proximal end of the fabric (Figure 4). Intraobserver and interobserver mean differences for proximal seal diameter were 0.23 mm (0.67%) and −1.94 mm (–5.66%), respectively.

For the distance to the end of the fabric, the mean intraobserver difference was −0.16 mm (–0.26%), while the interobserver difference was 2.32 mm (3.46%).

### Distal Seal Diameter and Distance to End of Fabric Measurements

For the diameter of the distal seal, intraobserver agreement demonstrated a mean difference of 0.40 mm (1.23%) across repeated measurements. To address heteroscedasticity in interobserver measurements, we applied a logarithmic transformation, resulting in a mean difference of 1.05 mm (2.91%).

**Figure 5.**
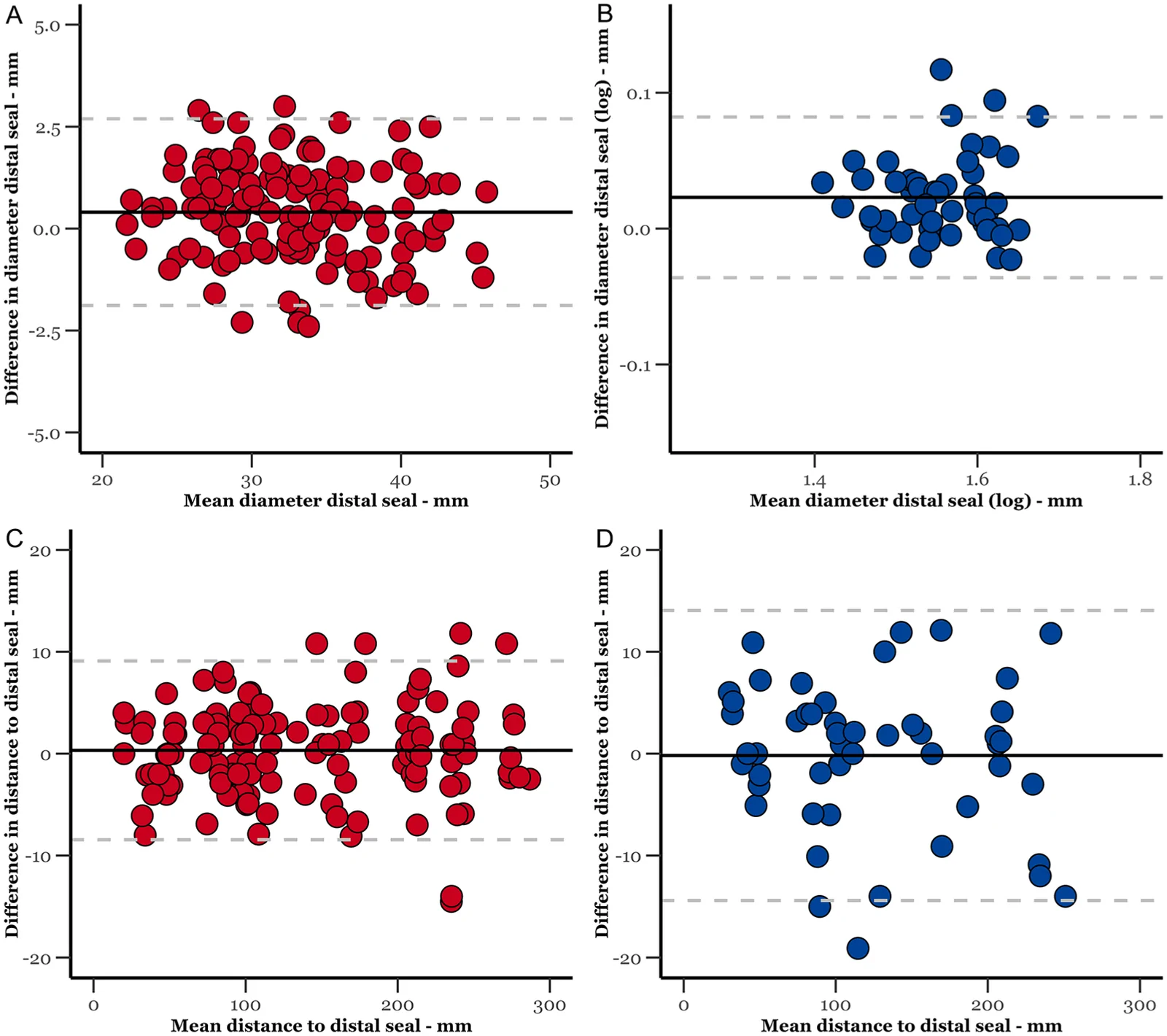
Bland-Altman plot of the intraobserver (in red) and interobserver (in blue) agreements for diameter and distance distal seal.

For the distance to the distal end of the fabric, intraobserver and interobserver agreements exhibited mean differences of 0.32 mm (0.23%) and −0.18 mm (–0.15%), respectively. The B-A plots are available in Figure 5.

### Length of Coverage and Aneurysm Length Measurements

The mean difference for length of coverage was 1.68 mm (1.07%) for intraobserver agreement and 6.35 mm (3.57%) for interobserver agreement. For the length of the TAA, intraobserver measurements showed a mean difference of 2.52 mm (0.67%), while interobserver measurements had a mean bias of 7.39 mm (1.93%). Corresponding B-A plots are depicted in Supplemental Figure S1.

### Length of Proximal and Distal Seal Measurements

The mean intraobserver difference for the length of the proximal seal was 0.84 mm (4.25%), while interobserver agreement showed a mean difference of 2.61 mm (13.44%).

For the length of the distal seal, the mean intraobserver difference was −0.02 mm (–0.09%) and the interobserver difference was 8.15 mm (29.80%). Corresponding B-A plots are presented in Supplemental Figure S2.

### Zones of Attachment

For both intraobserver and interobserver agreements in determining the sealing zones of attachment, all observations uniformly matched.

### Subgroup Analysis Computed Tomography Angiography Slice Thickness

Most scans had a low slice thickness of either 0.8 mm (n=44, 33%) or 1.0 mm (n=67, 50%), while fewer scans had high slice thickness of 1.5 mm (n=6, 5%), 2.0 mm (n=15, 11%), or 3.0 mm (n=1, 1%). No significant differences in intraobserver and interobserver measurement variabilities were observed between high and low CTA slice thickness (Supplemental Table S1).

### Intraclass Correlation for Intraobserver and Interobserver Agreements

For the intraobserver agreement, the results demonstrated perfect correlation for all 10 variables as evidenced by all ICC values >0.900 (Table 3). For the interobserver agreement, the results showed perfect correlation in most of the variables. Regarding the diameter of the proximal and distal seal, however, the ICC values indicated a moderate to perfect correlation. The length measurements indicated a fair to good correlation.

**Table 3.** The Intraclass Correlation (ICC) for Intraobserver and Interobserver Agreements.

	ICC (95% CI)	ICC interpretation
*Intraobserver agreement*		
Maximal diameter	0.984 (0.978, 0.989)	Perfect
TAA volume	0.996 (0.995, 0.997)	Perfect
Length of coverage	0.994 (0.988, 0.997)	Perfect
TAA length	0.987 (0.981, 0.991)	Perfect
Diameter proximal seal	0.962 (0.947, 0.973)	Perfect
Distance to proximal seal	0.992 (0.989, 0.994)	Perfect
Length of proximal seal	0.967 (0.952, 0.977)	Perfect
Diameter distal seal	0.975 (0.961 - 0.984)	Perfect
Distance to distal seal	0.998 (0.998, 0.999)	Perfect
Length of distal seal	0.988 (0.982, 0.991)	Perfect
*Interobserver agreement*		
Maximal diameter	0.976 (0.958, 0.986)	Perfect
TAA volume	0.993 (0.987, 0.996)	Perfect
Length of coverage	0.989 (0.930, 0.997)	Perfect
TAA length	0.982 (0.940, 0.992)	Perfect
Diameter proximal seal	0.908 (0.663, 0.963)	Good to perfect
Distance to proximal seal	0.988 (0.977, 0.993)	Perfect
Length of proximal seal	0.503 (0.268, 0.683)	Fair to good
Diameter distal seal	0.817 (0.509, 0.917)	Moderate to perfect
Distance to distal seal	0.994 (0.989, 0.997)	Perfect
Length of distal seal	0.771 (0.515, 0.884)	Moderate to good

Abbreviations: CI, confidence interval; ICC, intraclass correlation coefficient.

## Discussion

As with EVAR, morphological features are crucial in assessment of suitability of endovascular management and clinical outcomes after TEVAR.^20–22^ This underscores the importance of an accurate standardized protocol to improve consistency and avoid misleading conclusions with potential clinical significance. The lack of a standardized protocol has led to variability across institutions, as existing guidelines focus on reporting rather than standardizing measurement techniques.^5,6,9^ Such a protocol could minimize interobserver and intraobserver variabilities, enhance preoperative endograft sizing, support consistent imaging surveillance, and eventually facilitate the integration of AI-driven software’s, which relies on standardized measurements. In this study, we composed and internally evaluated the consistency of a CTA-based standardized measurement protocol following TEVAR for descending TAA. Our protocol demonstrated high agreement among most measurement sets, especially for measurements repeatedly taken by the same observer.

Considering the inherent variability in repeated measurements, a change of 5 mm in aneurysm diameter has traditionally been used as the threshold to define sac growth or regression.^7,23–25^ In our study, we found excellent intraobserver and interobserver agreements, and perfect correlation for maximal diameter measurements, indicating a strong consistency. This indicates that mean differences in diameter measurements, whether taken by the same observer or different observers (–0.45 and −0.33 mm, respectively), remained within the clinically important 5 mm threshold. Similarly, as the threshold for a significant volume change is 5% to 10%,^26–29^ our volume measurements displayed excellent agreement, remaining well below this threshold with minimal mean differences of 0.77% and 0.55%. The current thresholds for determining sac regression or growth are primarily based on historical variability in measurements between observers and across repeated measurements. Given the high consistency achieved using our newly developed protocol, this raises the question of whether the current thresholds for post-TEVAR surveillance should be re-evaluated. This is particularly of value in the absence of a standardized protocol until now. Although no established thresholds exist for the length of TAA,^7^ our findings of perfect correlation and high agreement in these measurements (0.67% and 1.93%), suggest that we can now potentially develop such thresholds, which could be of value in distinguishing true stent-graft migration and aortic elongation.^30,31^

Assessment of the distance from a fixed landmark to the proximal and distal seal, key metrics for stent-graft migration and angulation, revealed perfect correlation and high agreement within the significant 10 mm range.^7^ While measurements of the proximal and distal seal diameters showed slightly lower interobserver correlation, the mean differences were minimal. Importantly, averaging 2 perpendicular diameters addresses inaccuracies due to vessel tortuosity, offering a more reliable estimation.^15^ This method is efficient, making it a practical addition to routine clinical practice. Furthermore, while the length of coverage does not directly influence clinical decision-making, reporting it remains relevant as it is a known risk factor for paraplegia. Our protocol consistently measured the length of coverage, showing perfect correlation and minimal mean differences across measurements (1.07% and 3.57%). The determination of the zones of attachment, another proposed method to measure length of coverage, matched perfectly across all measurements. Our protocol thus offers reliable options for reporting length of coverage amidst the absence of a methodological consensus in the literature.^7^ Balancing length of coverage with adequate length of sealing is essential, as length of sealing is clinically more relevant, predicting endoleaks and long-term durability of TEVAR.^5,6^

In our study, the interobserver agreement was typically less reliable, possibly due to a smaller sample size in this cohort. However, it still exhibited acceptable levels of agreement in the measurements. Intraobserver and interobserver agreements concerning the length of the proximal and distal seals was less favorable (proximal seal: 4.25% and 13.44%; distal seal: –0.09% and 29.80%, respectively) compared with the length of TAA measurements. This discrepancy might stem from the more precise definition of TAA length using anatomical reference points, leading to reduced variability between observers. Furthermore, volume measurements seem to be a more sensitive early indicator of aneurysm growth than maximal diameter.^27,32–35^ In our study, volume measurements exhibited excellent intraobserver and interobserver agreements, along with an excellent correlation. This indicates that our protocol for measuring TAA sac volume is both consistent and reliable. This is important since this is a very strong predictor in survival and/or treatment success in patients with TAA after endovascular treatment.^36,37^

Similar to EVAR, fully automated AI tools for aortic segmentation have the potential to address the labor-intensive nature of semi-automated measurement protocols.^38–41^ For instance, volume measurements post-EVAR using the PRAEVAorta®2 software tool has demonstrated both accuracy and consistency.^13^ This is clinically relevant, as EVAR surveillance remains suboptimal, with the majority of patients not benefiting from surveillance imaging.^42–45^ A similar scenario might apply to post-TEVAR surveillance, although this remains unknown. Our standardized measurement protocol represents a foundational step toward enhancing imaging reporting consistency and possibly surveillance efficiency. Once externally and internationally validated, this protocol could facilitate the integration of AI tools, which rely on such standardization to maximize their utility. Artificial intelligence involvement could expedite the protocol’s validation by enabling the analysis of larger patient cohorts while ensuring consistent measurements across centers and observers. In the future, AI-driven software has the potential to revolutionize TEVAR surveillance, by supporting annual monitoring with greater quality, consistency, and efficiency. Enhanced detection of aneurysm sac regression or growth would likely improve post-TEVAR risk stratification. This advancement holds promise for both research and clinical practices. Therefore, we advocate for future studies to explore the consistency of AI tools in TEVAR surveillance, leveraging our proposed standardized measurement protocol.

Several limitations of this study need to be addressed. First, the proposed protocol relies on the 3mensio software for its user-friendly interface and accessibility at our center. However, the protocol might be adaptable to other software with necessary technical adjustments. Second, our protocol did not include measurements, such as angulation or tortuosity. Tortuosity is not routinely measured for preoperative sizing, but since the tortuosity index can be calculated as the ratio of TAA length to the straight-line distance between the same baselines,^12^ our high agreement of TAA length measurements provide a reliable foundation. Using the same baselines, the straight-line distance measurement should theoretically show similar consistency. Future studies should focus on angulation measurements. Third, our focus was exclusively on TAA, limiting the applicability of our findings and measurement protocol to other aortic pathologies, such as dissections, aneurysms involving the aortic arch branches or traumatic injury. Notably, the literature includes protocols tailored specifically for dissections, emphasizing the importance of condition-specific methodologies.^46^ Fourth, while a larger sample size could potentially detect smaller significant differences, we are confident that the current sample provided adequate statistical power for the conclusions presented. Despite the limited number of patients, the high number of CTA scans (n=133) and the fact that this is the first study to establish a TEVAR measurement protocol support its role as a proof of concept. Given the limitations of a post hoc power analysis, we did not perform one as various studies have demonstrated that one could be invalid and misleading and shall only complicate the interpretation of the analyses.^47–49^ However, for broad implementation of the protocol, more extensive validation in future studies involving larger patient cohorts and additional observers is necessary. Finally, our study included CTA scans with a maximum slice thickness of 3 mm, though this limitation does not necessarily prevent the protocol’s use with other imaging qualities. Only 17% of scans had higher slice thickness, which seemed to increase interobserver variability in just 2 measurements. Using uniformly high-quality CTA scans ensures accurate identification of anatomical landmarks and reduces possible confounders, facilitating a reliable assessment of the protocol’s consistency.

## Conclusion

Our standardized measurement protocol provides a consistent approach for assessing descending TAA characteristics following TEVAR, enhancing preoperative planning, imaging surveillance and research applications. This consistent methodology prompts the integration into AI-driven methods, which could make imaging reporting and surveillance more reliable and less time-consuming. Most measurements displayed high agreement, and agreement was especially higher in measurements repeatedly done by the same observer. Measurements of diameter, volume, and length displayed excellent consistency, which may initiate future reconsideration of the current thresholds for sac regression or growth in post-TEVAR surveillance. Future research should focus on external validation and study the potential integration of AI to further enhance the surveillance after TEVAR.

## Supplemental Material

sj-docx-3-jet-10.1177_15266028251380535 – Supplemental material for Feasibility and Validity of a Standardized Protocol for Measuring Thoracic Aortic Aneurysm Morphology Supplemental material, sj-docx-3-jet-10.1177_15266028251380535 for Feasibility and Validity of a Standardized Protocol for Measuring Thoracic Aortic Aneurysm Morphology by Olivier L.R.M. van Tongeren, Amber Sharman, Vinamr Rastogi, Rens B. Varkevisser, Sanne E. Hoeks, Arnoud V. Kamman, Hence J.M. Verhagen and Jorg L. de Bruin in Journal of Endovascular Therapy

sj-tiff-1-jet-10.1177_15266028251380535 – Supplemental material for Feasibility and Validity of a Standardized Protocol for Measuring Thoracic Aortic Aneurysm Morphology Supplemental material, sj-tiff-1-jet-10.1177_15266028251380535 for Feasibility and Validity of a Standardized Protocol for Measuring Thoracic Aortic Aneurysm Morphology by Olivier L.R.M. van Tongeren, Amber Sharman, Vinamr Rastogi, Rens B. Varkevisser, Sanne E. Hoeks, Arnoud V. Kamman, Hence J.M. Verhagen and Jorg L. de Bruin in Journal of Endovascular Therapy

sj-tiff-2-jet-10.1177_15266028251380535 – Supplemental material for Feasibility and Validity of a Standardized Protocol for Measuring Thoracic Aortic Aneurysm Morphology Supplemental material, sj-tiff-2-jet-10.1177_15266028251380535 for Feasibility and Validity of a Standardized Protocol for Measuring Thoracic Aortic Aneurysm Morphology by Olivier L.R.M. van Tongeren, Amber Sharman, Vinamr Rastogi, Rens B. Varkevisser, Sanne E. Hoeks, Arnoud V. Kamman, Hence J.M. Verhagen and Jorg L. de Bruin in Journal of Endovascular Therapy
