# Supplementary material for: Feasibility and Validity of a Standardized Protocol for Measuring Thoracic Aortic Aneurysm Morphology
Source: J Endovasc Ther. 2025 Oct 5;33(5):2677–88. doi: 10.1177/15266028251380535 (PMC13571004; doi:10.1177/15266028251380535)
Supplement: Supplementary file 1 [file sj-docx-3-jet-10.1177_15266028251380535.docx]

| Supplementary Table S1. Intra- and interobserver measurement differences by low (0.8 and 1.0 mm) vs. high (1.5, 2.0 and 3.0 mm) CTA slice thickness | | | | | | |
| --- | --- | --- | --- | --- | --- | --- |
| *Intra-observer* | |  |  | ***Inter-observer*** |  |  |
|  | | **Measurement difference** | ***p-value*** |  | **Measurement difference** | ***p-value*** |
|  | **Maximal diameter** |  | *.339* |  |  | *.490* |
|  | Low slice thickness | -0.4 |  |  | -0.2 |  |
|  | High slice thickness | -0.9 |  |  | -1.0 |  |
|  | **TAA volume** |  | *.285** |  |  | *.803* |
|  | Low slice thickness | -3.0 |  |  | 3.1 |  |
|  | High slice thickness | 4.8 |  |  | 0.2 |  |
|  | **Length of coverage** |  | *.808* |  |  | *.952* |
|  | Low slice thickness | 1.5 |  |  | 6.5 |  |
|  | High slice thickness | 2.5 |  |  | 6.1 |  |
|  | **TAA length** |  | *.899** |  |  | *.976** |
|  | Low slice thickness | 2.1 |  |  | 4.6 |  |
|  | High slice thickness | 2.1 |  |  | 2.8 |  |
|  | **Diameter proximal seal*** |  | *.246* |  |  | *.310** |
|  | Low slice thickness | 0 |  |  | -1.6 |  |
|  | High slice thickness | 0.6 |  |  | -2.8 |  |
|  | **Distance to proximal seal** |  | *.251** |  |  | *.681* |
|  | Low slice thickness | 0.1 |  |  | 2.6 |  |
|  | High slice thickness | 0.1 |  |  | 0.5 |  |
|  | **Length of proximal seal** |  | *.325* |  |  | *.109** |
|  | Low slice thickness | 1.0 |  |  | 3.7 |  |
|  | High slice thickness | 0.1 |  |  | -5.7 |  |
|  | **Diameter distal seal** |  | *.708* |  |  | *.199** |
|  | Low slice thickness | 0.4 |  |  | -2.1 |  |
|  | High slice thickness | 0.3 |  |  | -0.6 |  |
|  | **Distance to distal seal** |  | *.143* |  |  | *.361* |
|  | Low slice thickness | 0.6 |  |  | 0.2 |  |
|  | High slice thickness | -1.0 |  |  | -2.8 |  |
|  | **Length of distal seal** |  | *.198* |  |  | *.550** |
|  | Low slice thickness | -0.2 |  |  | 8.6 |  |
|  | High slice thickness | 0.8 |  |  | 5.0 |  |
| Measurement differences are expressed as means or medians, as appropriate  All analyses were performed using two-sample t test, or Mann-Whitney U test as indicated with *  *CI,* Confidence Interval; | | | | | | |
